# Barriers and implementation strategies for physical activity on prescription (PAP): healthcare personnel and management perspectives in Sweden—An explanatory sequential study design

**DOI:** 10.1186/s12875-025-03038-y

**Published:** 2025-10-06

**Authors:** Dan Sigvardsson, Anna MacLean, Albin Hoonk, Tahaa Ali, Marlene Makenzius

**Affiliations:** 1 The Department of Public Health, Kunskapens väg 8, Östersund, 83125 Sweden; 2 Department of Global Public Health, Karolinska institutet, Stockholm, Sweden

**Keywords:** Health promotion, Healthcare, Implementation, Leadership, Mixed-Methods design, Normalization process theory, Physical activity on prescription, Primary healthcare

## Abstract

**Background:**

Physical activity on prescription (PAP) is a recommended method in Swedish and European primary healthcare, yet implementation remains inconsistent. This study explored barriers and strategies for strengthening PAP use in a region with low prescription rates, from the perspective of healthcare personnel and managers.

**Methods:**

A mixed-methods study with an explanatory sequential design was conducted (2024) in a geographically dispersed Swedish region. Digital surveys with healthcare personnel and managers (*n* = 93) were analysed using uni-, bi- and multivariate analyses, followed by semistructured interviews (*n* = 14) analysed with qualitative content analysis guided by Normalization Process Theory (NPT).

**Results:**

The survey was completed by 75 healthcare personnel and 18 managers. Most healthcare personnel (84%, *n* = 58/69) reported providing basic PA advice regularly, but only 16% (*n* = 12/74) prescribed PAPs. Physiotherapists prescribed PAP more often than other personnel (32%, *n* = 8/25 vs. 8%, *n* = 4/47; *p* = .018), and this remained significant in adjusted analyses (OR 4.0, 95% CI 1.01–15.83). Documentation of PAP components according to the Classification of Health Care Interventions (KVÅ) was generally low. Physiotherapists more often reported high or very high competence in counselling compared with other healthcare personnel (100%, *n* = 27/27 vs. 72%, *n* = 34/47; *p* = .003), prescribing PAP (74%, *n* = 20/27 vs. 36%, *n* = 17/47; *p* = .003), and follow-up (78%, *n* = 21/27 vs. 32%, *n* = 15/47; *p* < .001). Female healthcare personnel expressed stronger trust in PAP evidence than males (*p* = .013). Few personnel had received PAP training (38%, *n* = 28/74), and most were unaware of existing guidelines (81%, *n* = 61/75). Time constraints (53%, *n* = 41/75) and lack of guidelines (48%, *n* = 38/75) were the most common barriers, with older healthcare personnel (≥ 46 years) more likely to report time constraints (OR 3.6, CI 1.27–10.16). Managers more often highlighted unclear purpose of PAPs and patient reluctance, whereas personnel emphasised lack of training and managerial prioritisation. Despite these differing perspectives, 69% (*n* = 50/72) of healthcare personnel and 75% (*n* = 12/16) of managers endorsed increasing PA counselling.

**Conclusions:**

Strong interest in PAPs indicates favourable conditions for sustainable implementation if legitimacy-building education (coherence), tailored resources and training (cognitive participation), multilevel leadership commitment (collective action), and coordination for monitoring (reflexive monitoring) are secured. These are necessary strategic investments, transferable beyond Sweden to systems facing PAP implementation challenges.

**Supplementary Information:**

The online version contains supplementary material available at 10.1186/s12875-025-03038-y.

## Background

Physical inactivity is a major contributor to the global burden of noncommunicable diseases (NCDs) and premature mortality [1]. The World Health Organization (WHO) attributed 20–30% of premature deaths to insufficient physical activity (PA) and estimated that 500 million preventable NCD cases may occur between 2020 and 2030 due to inactivity [1], with an associated annual healthcare cost of up to 27 billion US dollars [2]. Despite the WHO recommendations of 150–300 min of moderate PA per week [1, 3], over 25% of adults globally—and 34% in Sweden—fail to meet this target [3, 4].

Promoting PA is a core goal in both global and national health strategies and is a key component of Goal 3 of the UN’s Agenda 2030, which aims to ensure healthy lives and promote well-being for all ages [5]. In Sweden, PA is embedded in national public health goals related to lifestyle behaviours and health equity [6]. The Commission for Equity in Health [7] emphasised the need to strengthen individuals’ ability to promote health and prevent illness. The healthcare sector plays a central role in this effort, given its wide population reach and high credibility as a source of health information [8]. In 2021 alone, Swedish primary care recorded more than 36 million visits [9], highlighting its significant potential as an arena for preventive interventions.

The National Board of Health and Welfare (NBHW) in Sweden recommends the use of physical activity on prescription (PAP) for patients at risk owing to low levels of PA [10]. PAP includes individualised counselling, a written prescription, follow-up, and clinical guidance on the basis of the FYSS (Physical Activity in the Prevention and Treatment of Disease) handbook [11]. Collaboration with community-based activity providers is also encouraged. PAPs are currently implemented in all Swedish regions and are being adopted in other European countries [10, 11].

Evidence indicates that PAP can increase PA levels, improve quality of life [12–15], and offer long-term health and economic benefits [16]. However, prescription rates remain inconsistent across regions [11], and PAPs are often viewed more as a secondary measure than as part of standard care and a routine practice [17].

Several barriers hinder effective implementation, including unfamiliarity with PAPs, limited knowledge of their evidence base and preventive role, lack of managerial support, insufficient training, unclear routines, and time constraints [11, 18, 19]. Differences in regional support structures, training opportunities, and professional networks contribute to unequal delivery across the country [20].

As with other complex interventions, the implementation of PAPs is highly context dependent. Conditions that promote success in one setting may be ineffective—or even counterproductive—in another [21]. Context encompasses both structural factors (e.g., staffing levels, geographic location) and social dynamics (e.g., routines, leadership, interprofessional relationships) [22]. Implementation processes can, in turn, reshape these dynamics [22], and sustained success depends on behaviour change at both the individual and organisational levels [8].

Normalization process theory (NPT) provides a valuable framework for studying the implementation of complex interventions [23]. It focuses on how new practices become embedded in everyday clinical work [24] through four interrelated constructs: (i) coherence—how participants understand and make sense of the intervention, including its relevance and purpose; (ii) cognitive participation—the level of engagement and commitment among actors to adopt and sustain the practice; (iii) collective action—the operational work required to implement the practice, including task distribution and collaboration; and (iv) reflexive monitoring—the process through which individuals and teams assess effectiveness and adapt practices over time [24].

Many studies are limited by qualitative designs, small regional samples, and a focus on already motivated patients, which restricts generalisability and introduces selection bias. Research has also relied largely on patients’ perspectives, leaving a gap in knowledge about how healthcare personnel and managers view PAP, and how organisational context, staff competence, and leadership attitudes influence its delivery and long-term sustainability. Addressing these perspectives is essential for developing more effective and structurally supported implementation strategies [25].

Although PAPs are formally adopted by all 21 Swedish regions, decentralization has resulted in uneven implementation [11]. A sparsely populated region, located in mid-Sweden, has among the country’s lowest rates of PAP prescriptions [11]. Gaining a deeper understanding of barriers and facilitators in such underperforming contexts can inform more targeted and effective implementation strategies, both regionally and nationally.

This study aimed to identify barriers and potential mitigation strategies to strengthen PAP implementation, as perceived by healthcare personnel and managers in a rural region where primary healthcare services are geographically dispersed.

## Methods

This was a mixed-methods study with an explanatory sequential design [26] in a sparsely populated region in mid-Sweden during spring 2024. The quantitative survey data informed the subsequent qualitative interviews. The study was carried out in collaboration between Mid Sweden University and the regional public health department, and the reporting adhered to the STROBE [27] and COREQ [28] guidelines.

The study setting was Sweden’s third largest region by area, a sparsely populated region (~ 130,000 residents), with a mix of rural and urban communities. Approximately one-third of the population lives in rural areas. Healthcare services in this rural region are geographically dispersed, with limited staff and resources, and the long distances involved add further complexity to implementation efforts.

Licenced healthcare personnel authorized to prescribe PAPs (e.g., physicians, nurses, physiotherapists) in primary healthcare settings, including a habilitation clinic and a day psychiatry clinic, and their managers participated. Surveys (Additional files 1 and 2) were distributed via internal channels and reached approximately 140 out of approximately 350 eligible personnel and 20 managers out of 36. Reminder emails and calls were used to increase response rates. Interview informants were recruited through digital questionnaires, where participants could provide their contact details if they consented to take part in individual interviews.

This process yielded 14 informants (9 healthcare personnel and 5 managers), representing a range of roles and settings. Findings from the quantitative survey guided the focus of the subsequent qualitative interviews, allowing emerging patterns in prescribing practices and perceived barriers to be explored in greater depth.

All participants in the questionnaire surveys received written information about the study, its aim and voluntariness. Consent was obtained through completion of the questionnaire. Those who agreed to participate in interviews received both written and verbal information and provided informed consent. Respondents could withdraw at any time without giving a reason. To ensure confidentiality, no data were presented at the level of individual respondents or specific clinics. Results were reported only in aggregate form, and any potentially identifying details were removed or anonymised. To further safeguard confidentiality, we did not collect information on age or years in profession among the managers, as they constituted a small group.

Two structured online surveys were developed via Qualtrics software [29], one for healthcare personnel (36 items) and one for managers (16 items) (Additional files 1 and 2). The items included Likert scales, multiple-choice questions, and optional free-text responses. The surveys were refined through feedback from pilot testers (*n* = 13) and regional stakeholders. Of those reached, 75 personnel and 18 managers completed the surveys, yielding response rates of 54% and 90%, respectively.

Semistructured interviews (Additional files 3 and 4) were conducted with 14 volunteers via Microsoft Teams, which lasted between 32 and 43 min, with one exception in which the respondent was interrupted by work, resulting in a shorter duration of 21 min. The interviews were recorded, transcribed verbatim, and guided by the four domains of the NPT [23, 24]. Five interviewees also reviewed a summary of the preliminary survey results.

The quantitative data were analysed via SPSS (v29) via descriptive statistics, such as Pearson’s chi-square test, Fisher’s exact test, and binary logistic regression with odds ratios (ORs) and confidence intervals (CIs). A significance level of *p* <.05 was applied throughout. Qualitative data, including interview transcripts and open-ended survey responses, were analysed deductively via content analysis [30]. The analysis began with an initial reading of the transcripts to familiarize them with the data, after which meaning units were identified and coded according to the NPT constructs [23, 24]. Coding was performed by DS and then secondarily by MM. Discussions with the AM, AH, and TA were held to enhance credibility and ensure intercoder consistency, and discrepancies were resolved through further discussion.

The interdisciplinary research team included members with backgrounds in public health, medicine, nursing, habilitation, and geography. All the participants shared an interpretivist orientation and viewed healthcare as a complex adaptive system (CAS), recognizing that implementation efforts are influenced by dynamic interactions between system actors and contextual factors [21, 22]. Reflexive notes were kept throughout to support transparency.

## Results

The survey was completed by 93 participants, comprising 75 healthcare personnel and 18 managers, where a majority were women (75%), representing diverse professional backgrounds (Table 1).

**Table 1:** Survey respondent characteristics (*N* = 93)

Gender	*n*	%
Female	70	75.3
Male	20	21.5
Nonbinary/do not wish to disclose	3 (1/2)	3.2
Age (healthcare personnel only)		
20–35	14	18.7
36–45	23	30.6
46–55	22	29.3
55+	16	21.3
Profession		
Physiotherapist	27	29.0
Nurse	20	21.5
Doctor	15	16,1
Occupational therapist	5	5.4
Other	4	4,3
Manager	18	19.4
Years in current profession (healthcare personnel only)		
1–10	24	32.0
11–20	32	42.6
21+	19	25.3
Unit healthcare personnel		
Regional health centre	41	54.6
Private health centre	9	12.0
Psychiatry	19	25.3
Habilitation (adult/child)	6	8.0
Unit managers		
Regional health centre	14	77.8
Psychiatry and habilitation	4	22.2

While 84% (*n* = 58) of healthcare personnel reported providing basic PA advice monthly or more, only 16% (*n* = 12) prescribed PAPs regularly (Table 2).

Younger personnel (aged 20–35) were more likely to prescribe PAP compared to those ≥ 36 years (38% vs. 12%, *p* =.034). Physiotherapists prescribed PAP more often than other healthcare personnel (32% vs. 8%, *p* =.018). However, the age-related difference was not statistically significant in the adjusted logistic regression (OR 3.25, 95% CI 0.76–13.84, *p* =.111). Instead, profession was significant: physiotherapists were more likely than other healthcare personnel to prescribe PAP (OR 4.0, 95% CI 1.01–15.83, *p* =.048).

**Table 2:** Frequency of basic advice, counselling, and PAP prescription by profession (*n* = 72)

Profession	Basic advice		Counselling		Prescribes PAP	
	Once a year/never	Daily/weekly/monthly	Once a year/never	Daily/weekly/monthly	Once a year/never	Daily/weekly/monthly
Physician (*n* = 15)	2	13	2	13	14	1
Nurse (*n* = 20)	3	17	5	15	19	1
Physiotherapist (*n* = 27)	1	26	4	21	17	8
Psychologist (*n* = 4)	4	0	1	3	3	1
Occupational therapist (*n* = 5)	1	4	2	3	4	1
Other (*n* = 4)	1	3	0	3	3	0

Documentation of PA basic advice and counselling was generally low, with 82–88% (*n* = 61–65) of respondents (*n* = 74) reporting rarely or never documenting. Among those prescribing PAP at frequencies ranging from about once a year to daily (*n* = 32), 78% (*n* = 25) reported usually providing follow-up. Documentation of PAPs in medical records via Classification of Health Care Interventions Codes (KVÅ) was reported by PAP prescribers, with 31% (*n* = 10) documenting qualified counselling, 44% (*n* = 14) documenting PAP prescriptions, and 41% (*n* = 13) documenting follow-ups always or usually.

Documentation was more common among physiotherapists compared to other healthcare personnel, including always/often documenting qualified counselling (18% vs. 10%, *p* =.018), PAP prescription (40% vs. 6%, *p* <.001), and PAP follow-up (37% vs. 6%, *p* <.001).

### Perceived competence in paps

Self-reported competence in prescribing PAPs varies among healthcare personnel (*n* = 75). While most of the participants felt confident in providing basic advice or counselling, 50% rated their competence in prescribing PAPs as low/very low. Physiotherapists reported high/very high competence significantly more often than other healthcare personnel in counselling (100% vs. 72%, *p* =.003), qualified counselling (74% vs. 38%, *p* =.004), prescribing PAP (74% vs. 36%, *p* =.003), and follow-up of PAP prescriptions (78% vs. 32%, *p* <.001).

Few personnel had received PAP training (38%, *n* = 28), and 69% (*n* = 52) were unaware that training had previously been offered. Most of the personnel lacked access to guidance or support materials in their daily work, and 81% (*n* = 61) were unaware of the existing national short version of the PAP guidelines for clinics.

### Perceived barriers

PAP work consists of multiple components and is accompanied by various challenges, as demonstrated by the barriers identified in Table 3. Time constraints and a lack of guidelines were the two main barriers in both groups. Among the personnel, this was followed by a lack of knowledge among personnel and management, whereas manger reported an unclear purpose of PAPs and that patients do not want to have PAPs.

While perceived time constraints were commonly reported, a binary logistic regression indicated that personnel aged ≥ 46 years, compared with those aged ≤ 45 years, were more likely to perceive time constraints as barriers to PAP (OR 3.6, CI 1.27–10.16) when adjusted for gender and profession.

**Table 3:** Healthcare personnel’s perceived barriers and suggested implementation measures for PAP adoption

Perceived barriers and mitigating strategies		Total (*N* = 93)	Healthcare personnel (*n* = 75)	Managers (*n* = 18)	Fisher’s exact test
Barriers		n (%)	n (%)	n (%)	
Perceived time constraints	49 (52.6)	41 (54.6)	8 (44.4)	0.60	
Lack of guidelines on PAP procedures et cetera within the region	45 (48.4)	38 (50.7)	7 (38.9)	0.437	
Lack of knowledge among healthcare personnel	40 (43.0)	37 (49.3)	3 (16.7)	.016	
Lack of knowledge/interest from management	36 (38.7)	34 (45.3)	2 (11.1)	0.007	
Unclear purpose of PAP	24 (25.8)	17 (22.7)	7 (38.9)	0.228	
Patient not receptive to/do not want PAP	21 (22.6)	15 (20.0)	6 (33.3)	0.227	
Lack of financial incentives	14 (15.0)	12 (16.0)	2 (11.1)	1.0	
Technical barriers	7 (7.5)	7 (9.3)	0 (-)	0.339	
I have no opinion	9 (9.7)	7 (9.3)	2 (11.1)	1.0	
Potential mitigating measures					
Educate more healthcare personnel in the PAP method	54 (58.0)	48 (64.0)	6 (33.3)	0.031	
Increase knowledge about PA/PAP among the population	41 (44.0)	32 (42.7)	9 (50)	0.606	
Management needs to prioritize PAP	35 (37.7)	32 (42.7)	3 (16.7)	0.041	
Improve access to information about PAP for personnel (e.g. on the region’s website)	35 (37.7)	29 (38.7)	6 (33.3)	0.790	
Increased collaboration on PAP	27 (29.0)	22 (29.3)	5 (27.8)	1.0	
Simplify the systems (such as documentation)	25 (26.9)	22 (29.3)	3 (16.7)	0.380	
Possibility to delegate follow-up of PAP to someone other than prescriber	22 (23.6)	20 (26.7)	2 (11)	0.224	
Financial incentives for PAP	22 (23.6)	16 (21.3)	6 (33.3)	0.355	
I have no opinion	8 (8.6)	4 (5.3)	4 (22.2)	0.043	

### Potential mitigating measures

The most frequently suggested mitigating measure to improve PAP work was to educate more healthcare personnel on this method (Table 3). It was proposed by 64% of personnel but only 33% of managers. Personnel were also more likely than managers to cite the need for management to prioritize PAPs (43% vs. 17%). Approximately half of both personnel and managers highlighted the need to raise public awareness of PAPs. Additionally, approximately one-third of both groups emphasized the need to improve personnel access to information about PAPs and to enhance collaboration in their implementation (Table 3).

More than half of healthcare personnel (52%, *n* = 39) expressed a need for improved patient materials in plain Swedish for groups with special needs, and 59% (*n* = 44) wanted materials available in other languages. A large majority (80%, *n* = 60) highlighted the need for collaboration with community activity providers, such as gyms and sports clubs, whereas 84% (*n* = 63) believed that a consolidated list of home-based activity suggestions would support PAP work.

Among respondents (healthcare personnels and managers), 54% (*n* = 50) perceived strong/very strong evidence for PAP as a method to promote health, while 14% (*n* = 13) rated the evidence as poor/very poor and 30% (*n* = 28) had no opinion. For PAP as an effective treatment or part of treatment, 51% (*n* = 46) perceived strong/very strong evidence, 12% (*n* = 11) rated it as poor/very poor, and 37% (*n* = 34) had no opinion. Female healthcare personnel were more likely than male personnel to express trust in evidence for PAP (*n* = 36/41, 88% vs. *n* = 8/14, 57%; *p* =.013).

Despite the identified barriers and the low number of PAP prescriptions 69% (*n* = 50) of healthcare personnel expressed a desire to increase their commitment in PA counselling. Similarly, 75% (*n* = 12) of managers endorsed an increased emphasis on PA counselling among their personnel.

### Qualitative findings

The qualitative analysis began by categorizing the open-ended responses from the survey into the NPT domains. The overall findings from the qualitative analysis revealed four themes (Table 4).

**Table 4:** Interview findings illustrating themes on barriers to PAP work and corresponding implementation solutions (healthcare personnel = 9; managers = 5)

Domain of NPT	Code	Category	Theme
Coherence	*Strong/poor evidence for PAP*	Lack of consensus regarding PAP as a method	Need for legitimacy-building training activities that create consensus
	*Meeting patient expectations*		
	*Lack of anchoring in scope of practice/Statutory role description*	Lack of formal responsibility	
	*Responsibility*		
Cognitive participation	*Praxis norms vary*	Generalization and short-sightedness	Need for adapted materials and routines to foster engagement and participation
	*Need for adapted routines and guidelines*		
	*Projects hinder long-term implementation*		
	*Health information/education*	“It takes two to tango” in patient encounter	
	*Sense of competence*		
Collective action	*Systematization and formalization*	Structural organization	Need for clear leadership and resources for a collectively sustainable PAP work
	*Cross-sector collaboration*		
	*Financial incentives*	Finding the right tools and support	
	*Motivation-building*		
	*Overcome time- and resource shortages*		
	*Support resources*		
	*Technical solutions*		
Reflexive monitoring/evaluation	*Heterogeneous group working across various healthcare settings*	Unsynchronized PAP work reflected in cognitive participation and collective action	PAP work needs to be organized at different levels for systematic monitoring/evaluation
	*Rural perspective as a complicating factor*		
	*Divergent views of benefits*		
	*Low/uneven PAP prescription rates between groups*		

### Need for legitimacy-building training activities that create consensus

The theme covers the coherence domain of the NPT and encompasses patient expectations, awareness and a lack of consensus on PAPs.

The participants described inconsistent views about PAPs’ relevance and effectiveness. Some perceived PAPs as overlapping with routine PA advice and counselling and questioned the added value. Doubts about patient receptivity were common:

“…*they [patients] want the doctor’s note for sick leave and are not prepared to do much… They’ve already decided beforehand.”* (Manager 2).

Prescribing PAPs was also argued to be potentially uncomfortable for the prescriber, since patients could end up feeling insulted: “*If it is a smoker or overweight*,* and then some fitness fanatic says you should exercise more*,* of course some might find it a bit of an invasion of privacy*” (Healthcare personnel 6).

Managers also described low awareness of PAPs within their teams. 

Manager 2 expressed “*In my workplace*,* I think we talk too little about PAPs. In addition*,* overall*,* we probably know too little about it… we would need to highlight it in a clearer way*,* that’s how I feel self-critically.*”

Meeting educational needs was considered a potential solution. Manager 4 argued that “*… it’s about educational initiatives…*” while another described how a course in PAP had contributed to increased motivation among participants who “…*have been quite motivated*,* those who have participated in it now.”* (Manager 5).

The educational need was also argued to extend to the patients themselves, who needed to be better informed of the existence of PAPs:

“*I think it’s a lack of knowledge among citizens*,* our patients. It might be them who need to be informed that it exists and that it could be good for them…*” (Manager 1).

The respondents specifically expressed a lack of consensus about PAPs’ added value and use. Healthcare personnel 1 said, “*That piece of paper is not so important for us in our clinical everyday work. More importantly*,* we have informed the patient that this is something you need to start doing.*”

### Need for adapted materials and routines to foster engagement and participation

The theme covers the cognitive participation domain of the NPT, encompasses perceptions of PAPs as discontinued projects, and calls for adapted routines and clear guidelines.

The respondents described PAPs as methods that had lost momentum over time, with several referring to PAPs as “forgotten projects.” Despite initial efforts to introduce PAPs, the lack of continued emphasis or integration into care processes meant that PAPs were often sidelined. This was especially common among managers, which was argued to have led to an overall loss of focus on PAPs. One manager described that:

“*I think there was a red thread in the introduction of it*,* maybe eight years ago… Eh*,* but somewhere along the way… then the momentum was lost and the management in it*,* just like with so many projects… It’s a bit like ‘well*,* that just sort of fell through the cracks*,* that didn’t truly become anything’…*” (Manager 5).

The absence of discussion about PAPs in some units was initially interpreted by managers as evidence of successful integration.

“*Well*,* you know*,* since I came here*,* I’ve never heard of PAPs being discussed. Eh*,* then I thought maybe it is because it’s a well-established method… so that is why it’s not talked about in the same way. However*,* I realized later when this survey came*,* and I started asking around*,* that for us*,* it is only the physiotherapists who prescribe it.*” (Manager 1).

The lack of explicit guidelines and structured follow-up further hindered engagement. The participants noted that although physical activity was sometimes mentioned in care programs, PAP specifically was not mentioned, leading to vague and inconsistent use.

*“It’s not that it’s stated that it’s included with various diseases and so on*,* in the care programmes. PA is mentioned*,* but not specifically PAP*,* and then it just becomes this little… bit of advice… It’s not truly followed up*.” (Manager 5).

Without integration into existing workflows and follow-up structures, PAPs remained the responsibility of enthusiastic individuals rather than part of standard care. Several interviewees called for clearer procedures and embedded routines to foster sustained engagement.

### Need for clear leadership and resources for collectively sustainable PAP work

The theme covers the collective action domain of the NPT and encompasses an identified need for leadership and support, perceptions of motivation-building work, and technical solutions to and systematization of PAP work.

Leadership and organizational support emerged as central to successful implementation. Many participants reported that PAPs were not actively promoted by managers, leaving them to individual staff to seek out information or take initiative. One respondent explained that “*PAP is not something the managers have encouraged*.” (Healthcare personnel 9).

Others emphasized the importance of role models, peer networks and accessible online resources for embedding PAPs and sustaining future efforts across geographically dispersed healthcare settings. “*If the region could provide something… great examples from others. It should be simple. Not time-consuming. Because if it becomes time-consuming*,* it gets set aside*,* that is how it is.”* (Manager 2). The need for easy access to information was a recurring view. Manager 3 said, *“… you want it to be easy to find quickly. In addition*,* I don’t think the region’s website is very well known for being able to quickly find what you need*.”

Support was also described as having the right people in the right place, for example by engaging a motivated colleague to act as an ambassador and coordinator. Technical solutions was also suggested to simplify documentation system or a PAP app for patients were proposed to reduce administrative burden.

“*I can picture an app where the patient reports their physical activity… I think it would be a bit like self-monitoring blood pressure. You might get a list of those who are in the red or orange for that week*,* from those you have prescribed to*,* and then you can quickly*,* like*,* send a little chat or.*.” (Manager 5).

A clear consensus was also that PAP work needs to be systematized and formalized. For example, PAPs could be used as leverage in patient meetings if there are, e.g., financial incentives for patients, such as discounts at gyms.

“ *It could motivate the personnel to prescribe more. Because they could motivate by saying… well*,* look*,* you get to try it for free*,* or you get a discount if you sign up for a training card there*.” (Manager 3).

At a structural level, the need for designated PAP coordinators within each healthcare unit, as well as at the regional level, was viewed as crucial for continuity and strategic leadership. These individuals could serve as resource persons, organize training, and facilitate collaboration across sites. Manager 5 said, “*There should be someone responsible for PAPs*,* who has some special training and is a support for other personnel. In addition*,* then a network for them*,* so they can collaborate in the region and get ideas from each other.*”

Staff turnover was often cited as a barrier to the continuation of PAPs. Manager 5 explained, “*Then*,* it is difficult for us*,* with so many locum doctors*,* who also have different experiences*,* and… you use it a bit differently. Therefore*,* there should be clearer guidelines there.*” A lack of both time and resources further increased PAPs being deprioritized. “*It truly is time scarcity. It feels like we’re always complaining about time*,* but that’s unfortunately how it is. Even though we*,* as physiotherapists*,* still have quite a bit of time for a first visit compared to doctors and nurses*,* it can still be difficult to manage writing out PAP*,* and that’s the challenge.*” (Healthcare personnel 3). Additionally, managers saw time scarcity as a critical factor: *“…PAP is just one of those things that gets lost when you’re busy trying to get staffing for doctors and nurses and making the day run smoothly*,* then it’s not a priority*,* for the day at least…*” (Manager 5).

Personnel also emphasized the difficulty of prioritizing PAPs amid competing demands. Healthcare personnel 5 said “*Yeah*,* I’d need 300% or 500% working time to be able to implement everything that’s cost-effective… We still must prioritize these things. What brings the most benefit?*”

### PAP work needs to be organized at different levels for systematic monitoring/evaluation

The theme covers the reflexive monitoring domain of the NPT. Few settings track PAP use, and without clear feedback mechanisms, it is difficult for personnel to assess the effectiveness or feel ownership of the process.

Barriers to reflexive monitoring were closely linked to earlier themes—limited awareness and consensus on PAPs, a varied view of the utility of PAPs, unclear routines, and insufficient systematization/formalization of PAPs—making it difficult for healthcare personnel to assess whether their efforts were effective or warranted. However, the participants offered suggestions on how to redefine procedures or modify practices to make them more workable in practice. Coordinated training for healthcare personnel, prioritization of PAPs in internal policies, and clearer integration into care workflows were all seen as potential strategies to embed monitoring more effectively. Healthcare personnel 1 framed the educational need as follows: “*We’ve studied during a time when this was launched. When older employees studied*,* they did not discuss it in the same way. Therefore*,* it is a newer concept that’s come up recently*,* and younger people tend to pick it up through their education.*”

Most respondents argued that PAPs need to be better prioritized but also that there is a need for broader structural integration of PAPs—into public health initiatives, regional health plans, and incentive frameworks such as the “health choice” (hälsovalet) —for long-term sustainability. Manager 2 stated that “*If this is something that should be prioritized*,* it’s also important what’s communicated to regional management above the local manager. Things that aren’t included in health choices tend to be less prioritized currently*.”

Patients were also seen as playing a role in normalization. Several respondents noted that broader public health communication could help generate demand “from below,” shifting the dynamic from one of professional push to patient pull.

“ *When it spreads*,* and the citizens demand it themselves*,* it creates a different momentum… Rather than sitting there and trying… Yeah*,* well*,* have you heard about this?*” (Manager 1).

## Discussion

This study aimed to identify challenges and propose strategies and measures to strengthen the implementation of the PAP method from the perspectives of both healthcare personnel and managers. The findings reveal considerable gaps between policy intentions and everyday practices. While the quantitative data illuminated patterns of usage, attitudes, and organizational prerequisites, the qualitative findings provided depth and context, offering an understanding of the mechanisms that influence whether and how PAPs become part of routine care.

While most healthcare personnel regularly provide basic advice on PAs, only 13% prescribe PAPs monthly, and documentation was rare. Compared with the other groups, physiotherapists were more likely to prescribe PAP and reported higher perceived competence in the method.

Major barriers included time constraints, unclear procedures, limited training, inconsistent leadership, a lack of collaboration, a lack of consensus over the evidence base and usefulness of PAPs, and a lack of prioritization. Despite these challenges, there was strong interest among both personnel and managers in expanding PA counselling efforts. However, female personnel had greater trust in PAP, which may reflect gendered differences in professional practice. Prior studies show that female healthcare providers are more likely to deliver preventive counselling and adopt patient-centred communication styles, both of which align with the principles of PAP [31].

These findings align with previous research [11, 18–20]. Despite national endorsement, PAPs are not yet fully integrated into Swedish routine healthcare [11], and in the current study, most healthcare personnel and managers expressed a desire for better conditions to work with this method.

### Need for legitimacy-building training activities that create consensus (coherence)

The qualitative findings confirmed that PAPs lack formal anchoring within healthcare settings. Many respondents perceived PAPs as tools that lacked added value, and some even questioned their appropriateness in certain patient interactions, fearing that they might feel intrusive or judgmental. This lack of consensus on the role and utility of PAPs undermines their legitimacy and signals a need for coordinated legitimacy-building activities. Both healthcare personnel and managers requested improved regional and cross-sectoral collaboration with PAPs. Gustavsson et al. [8] also stressed the importance of both a regional coordination function and local key players at the clinic level to support PAP efforts.

### Need for clear leadership and resources for a collectively sustainable PAP work (collective action)

Staff turnover was also cited as a persistent barrier related to the need to retrain new staff while dealing with staff shortages. Similarly, time constraints were considered critical barriers by both managers and healthcare personnel, and many respondents indicated that PAPs were simply deprioritized in the face of other, more immediate demands. This is consistent with earlier studies highlighting that competing demands on staff time and operational pressures are significant challenges to the sustainability of health promotion interventions in clinical practice [32, 33]. Interestingly, older healthcare personnel more often reported time constraints for PAP work, possibly reflecting differences in educational exposure to the method. However, Brorsson Lundqvist et al. [18] reported that more experienced physicians (>10 years) were more likely to prescribe PAP than their less experienced colleagues were. Addressing staff turnover and time constraints through adequate managerial support, clear routines and ensuring time and resources to train staff as needed, not only at the beginning of implementation, could help integrate PAPs into daily practice.

Another frequently cited barrier was the perceived unclear purpose of PAPs and perceptions of patients not being receptive to PAPs, particularly among managers. Healthcare personnel, meanwhile, highlighted internal barriers such as a lack of procedures and managerial support, as well as low competence. Few personnel were even aware of which colleagues were working with PAPs in their setting. These differing perceptions underscore the need for structured dialogue between management and personnel to align strategies and expectations, as suggested by Guerrero et al. (2020).

In addition, Guerro et al. demonstrate that effective implementation leadership depends on alignment across leadership levels, where transformational vision from top managers is translated into daily practice by middle managers—an insight directly relevant for embedding PAP through coherence, collective action, and sustained organisational support [34].

### Need for adapted materials and routines to foster engagement and participation (cognitive participation)

On the other hand, the study identified several opportunities to strengthen PAP efforts. Most healthcare personnel showed confidence in PAPs’ benefits and expressed a willingness to expand PA counselling. Access to simplified routines, ready-to-use patient materials (including in plain language and multiple languages), and improved collaboration with community activity providers were suggested as pragmatic next steps. Magi et al. (2024) underline the importance of patient materials that strengthen health literacy and self-care, which is vital for adherence to PAP. Uchmanowicz et al. (2025) show that integrating preventive interventions such as frailty care into routine care models enhances outcomes and sustainability. Their work reminds us that frailty care should not be overlooked when developing PAP strategies, as balancing acute and preventive needs is essential. These findings [35, 36] reinforces our finding that sustainable PAP requires both patient-centred resources and organisational integration.

Our findings also highlight the need for greater integration of PAPs into public health strategies and for regional-level initiatives to foster collaboration with external providers, such as municipalities and community activity providers. This is particularly important in rural areas, where access to activities may be limited, making such collaborations even more crucial. The inclusion of PAP indicators in internal performance goals could further strengthen its role in routine care.

### PAP work needs to be organised at different levels for systematic monitoring and evaluation (reflexive monitoring)

Finally, variations in perceptions of PAPs’ evidence base suggest a need for targeted training. While some personnel were sceptical about the method’s long-term effectiveness, others expressed disbelief in its usefulness compared with other forms of PA counselling. Although evidence supports PAPs’ ability to increase self-reported PA levels [16, 37], uncertainties remain, especially regarding sustained behavioural change [38]. Training efforts should therefore present a balanced view, acknowledging both the strengths and limitations of the method.

### Strengths and limitations

A key strength of this study is its use of a mixed-methods sequential design, which combines quantitative data with interviews to generate a rich understanding of PAP implementation [26]. Collaboration with regional stakeholders enhanced the study’s contextual relevance.

The relatively small sample size may limit statistical power and generalisability, and participation bias cannot be ruled out, as both those supportive of PAP and those critical of it may have been more inclined to respond. Nonetheless, the presence of both perspectives strengthens the credibility of the findings. Future research should include patient perspectives, adopt longitudinal designs, and use larger, more systematically selected samples with higher response rates to confirm and extend these results. Despite these limitations, the study provides valuable insights into current trends and barriers in PAP prescription among health care providers and managers in primary care within this region.

### Implications

This study underscores the importance of aligning priorities between healthcare personnel and management to strengthen PAP implementation. Embedding PAPs in routine practice requires leadership support, adequate training, and a shared understanding of their goals and benefits. Furthermore, fostering collaboration with community-based actors is crucial, especially in rural settings where healthcare resources are more limited. Technical tools and infrastructure must support—not hinder—the work of staff. Finally, evaluation mechanisms must be implemented so that PAP practices can evolve on the basis of feedback and real-world effectiveness. Efforts to implement PAPs will likely remain fragmented unless they are integrated into broader systems of accountability and public health promotion. Aligning PAPs with local care plans, incentive systems, and regional goals may provide the structural support needed for long-term success. These insights can inform strategies to integrate PAPs more effectively, both in Sweden and in other countries seeking to address physical inactivity and promote health equity.

## Conclusions

In this study, PAPs were widely regarded as evidence-based and relevant by both healthcare personnel and managers, yet their use was inconsistent and poorly integrated into routine practice. The key barriers included (i) time constraints, (ii) a lack of shared understanding, (iii) weak engagement, (iv) insufficient leadership, and (v) minimal systematic follow-up. These challenges highlight that simply introducing an evidence-based method is not enough; success depends on shared understanding, clear roles, and active support within the healthcare system.

Despite these challenges, strong interest in PAPs among healthcare staff and managers indicates favourable conditions for sustainable implementation if the core mechanisms for normalisation are secured. Coherence requires a shared fundamental understanding of PAPs, supported by accessible patient materials that reinforce communication with patients. Cognitive participation is strengthened through training that translates knowledge into practice and by local PAP coordinators who sustain engagement. Collective action depends on clear structures for all health personnel groups, robust internal and external networks, and collaboration with community providers. Leadership ensures legitimacy by allocating time and resources within supportive organisational frameworks. These measures should be regarded as necessary long-term investments rather than temporarily or optional recommendations. Moreover, the findings are transferable beyond Sweden to other health systems where PAP continue to face challenges in uptake and sustainability.

## Supplementary Information

Supplementary Material 1.

Supplementary Material 2.

Supplementary Material 3.

Supplementary Material 4.

## Data Availability

The datasets generated and/or analysed during the current study are available from the corresponding authors upon reasonable request.

## Abbreviations

FYSS: Physical activity in the prevention and treatment of disease (Fysisk aktivitet i sjukdomsprevention och sjukdomsbehandling)
NPT: normalization process theory
PA: Physical activity
PAP: Physical activity on prescription
KVÅ: Klassificering av vårdåtgärder (classification codes of Health interventions)
