# Supplementary material for: Barriers and implementation strategies for physical activity on prescription (PAP): healthcare personnel and management perspectives in Sweden—An explanatory sequential study design
Source: BMC Prim Care. 2025 Oct 6;26:302. doi: 10.1186/s12875-025-03038-y (PMC12502212; doi:10.1186/s12875-025-03038-y)
Supplement: Supplementary file 1 — Supplementary Material 1. [file 12875_2025_3038_MOESM1_ESM.docx]

# Interview Guide Healthcare Personnel 2024-04-18

## Background

Interview no.:

Date:

Interview duration:

Interviewer:

Age (reported as range xx–xx):

Gender:

Job title:

Years in current service (reported as range xx–xx):

All results are reported at group level; no individual results are presented linked to the above.

## Question 1

According to the survey sent out to healthcare personnels in your region, the majority believed there is good research evidence supporting PAP as a method. Despite this, most reported that they rarely prescribe PAP.

• What is your perception of why this is the case (providing advice on physical activity but not prescribing PAP)?

• Please describe further what might explain this situation.

## Question 2

The results showed that about half of the healthcare personnels rated their competence in prescribing PAP as low or not at all good.

• What do you think this is due to?

• What do you think is needed to strengthen staff competence?

## Question 3

You’ve now shared some of your thoughts on competence around PAP. You rgion prescribes fewer PAPs compared to other regions.

• Is it only about competence, or could there be other reasons? What are your thoughts?

• The results showed that younger staff are more inclined to work with PAP than older staff. Why do you think that is?

## Question 4

How do you and your colleagues view PAP as a method to promote physical activity?

• How do your colleagues perceive it, in your opinion? (Effectiveness? Usability?)

• How do you view your role and its impact on your colleagues’ perceptions of PAP?

• If you believe PAP should be implemented to a greater extent, what can you contribute to initiating that work? (Who/what else should be involved?)

## Question 5

How do you view collaboration within your unit, the region, and with other local/regional actors?

• For example, results show that most did not exchange experiences regarding PAP at their workplaces. What do you think is the reason?

• What is your view on local/regional networks within PAP? What can they contribute or potentially contribute? (e.g., benefits, content)

• How should a network be designed to suit/develop your unit?

• How would you like collaboration to look?

- Locally, regionally

- External collaboration partners

• Your region is a sparsely populated county. Does that affect PAP work? If so, how and why?

• How can PAP work be facilitated from a rural perspective?

## Question 6

Questionnaire results indicated that many felt that management did not prioritize PAP work.

• What is your opinion on this?

• What role do managers have in PAP work?

• How do you view your opportunities as a staff and your willingness to prioritize PAP work, compared to other tasks?

• How do you think PAP work needs to be organized? (That is, if you believe organization is needed to increase the number of PAPs)

## Question 7

• What do you think PAP work will look like in 10 years?

Is there anything else you would like to add?
