# Supplementary material for: Barriers and implementation strategies for physical activity on prescription (PAP): healthcare personnel and management perspectives in Sweden—An explanatory sequential study design
Source: BMC Prim Care. 2025 Oct 6;26:302. doi: 10.1186/s12875-025-03038-y (PMC12502212; doi:10.1186/s12875-025-03038-y)
Supplement: Supplementary file 2 — Supplementary Material 2. [file 12875_2025_3038_MOESM2_ESM.docx]

# Survey Questionnaire Healthcare Personnel – English Translation

## Q1

By answering this questionnaire, you confirm that you have read the study information and consent to participate in the study.

## Q2

Gender identity

o Woman (1)
o Man (2)
o Non-binary (3)
o Prefer not to say (4)

## Q3

Age

o 20–35 (1)
o 36–45 (2)
o 46–55 (3)
o 55+ (4)

## Q4

Professional role

o Physician (1)
o Nurse (all specialties) (2)
o Physiotherapist (3)
o Occupational therapist (5)
o Psychologist (4)
o Other (6)

## Q5

Years of professional experience in your current role

o 1–10 (1)
o 11–20 (2)
o 21+ (3)

## Q6

Which type of practice do you work in?

o Public health center (1)
o Private health center (2)
o Psychiatry (3)
o Adult or child rehabilitation (4)

How often do you give advice on physical activity and prescribe PAP?

Daily (4) / Weekly (5) / Monthly (6) / Yearly or never (8)

How often do you give simple advice (e.g., how physical activity affects health, encouraging increased activity, reducing sedentary behavior)?

How often do you give counseling (e.g., longer conversations with individually tailored advice)?

How often do you prescribe PAP?

**Q8**

Are patients followed up after receiving a PAP?

o Yes, most often (1)
o Rarely or never (2)
o I do not prescribe PAP (3)

**Q9**

Who follows up with the patient after a PAP prescription? Multiple options possible.

▢ Myself (1)
▢ Someone else (e.g., delegated to another healthcare professional such as a district nurse, physiotherapist, or PAP-trained assistant nurse) (2)
▢ The patient is not followed up (3)
▢ I do not prescribe PAP (4)

**Q10**

How often do you document KVÅ codes in the patient record (advice, PAP prescription, and follow-up)?

Always (1) / Often (2) / Rarely (3) / Never (4)

Simple advice (KVÅ code: DV131)

Extended counseling (KVÅ code: DV132)

Qualified counseling (KVÅ code: DV133)

PAP prescription (KVÅ code: DV200)

PAP follow-up (KVÅ code: DV500)

**Q11**

Is it clear when each KVÅ code should be used?

o Yes (1)
o Partly (2)
o No (3)
o No opinion (4)

**Q12**

If you find any KVÅ codes unclear, please comment or specify which ones you find unclear:

________________________________________________________________

## Q13

How do you assess your competence in physical activity to:

Very high(1) / high(2) / Low (3) / Very low (4)

Provide simple advice (e.g., how physical activity affects health, encourage more activity, reduce sedentary behavior)?

Provide counseling (e.g., longer sessions with individually tailored advice)?

Provide qualified counseling (often several and longer sessions using appropriate methods and subject expertise)?

Prescribe PAP?

Follow up on PAP prescriptions?

## Q14

Have you received any training in the PAP method beyond your professional education?

o Yes (1)
o No (2)

## Q15

Have you received any training in counseling techniques (e.g., Motivational Interviewing) beyond your basic professional education?

o Yes (1)
o No (2)

## Q16

What is your opinion of the research evidence supporting PAP as a health-promoting method?

o Very strong evidence (1)
o Strong evidence (2)
o Limited evidence (3)
o No evidence (4)
o No opinion (5)

## Q17

What is your opinion of the research evidence for PAP as a treatment or part of treatment for certain illnesses?

o Very strong evidence (1)
o Strong evidence (2)
o Limited evidence (3)
o No evidence (4)
o No opinion (5)

## Q18

In Jan/Feb 2024, PAP training invitations were sent to healthcare staff in your region. How do you perceive the availability of such training in previous years?

o Sufficient (1)
o Insufficient (2)
o Don't know (3)

## Q19

How do you perceive access to guidelines, advice, and support for prescribing PAP in your daily work?

o Very good (1)
o Good (2)
o Less good (3)
o Not good at all (4)
o No opinion (5)

## Q20

How is PAP work prioritized at your workplace?

o High priority (1)
o Moderate priority (2)
o Low priority (3)
o No opinion (4)

## Q21

How effective do you perceive PAP to be relative to the time it takes for healthcare staff?

o Very effective (1)
o Effective (2)
o Ineffective (3)
o Very ineffective (4)
o I do not prescribe PAP (5)

## Q22

Do you know who actively works with PAP at your workplace?

o Yes, good knowledge (1)
o Some knowledge (2)
o No knowledge (3)
o No one works actively with PAP (4)

## Q23

How do you perceive the exchange of experiences regarding PAP among your colleagues?

o Valuable (1)
o Somewhat valuable (2)
o Not valuable (3)
o No exchange of experiences (4)
o No opinion (5)

## Q24

Are you active in any PAP network? Multiple answers possible.

▢ Yes, locally (1)
▢ Yes, regionally (2)
▢ Yes, nationally (3)
▢ Yes, internationally (4)
▢ No (5)

## Q25

How do you perceive patients' receptiveness/attitude towards PAP?

o Very positive (1)
o Positive (2)
o Somewhat negative (3)
o Very negative (4)
o No opinion (5)

## Q26

Do you see a need for patient materials on physical activity/PAP in simplified Swedish?

o Yes (1)
o No (2)

## Q27

Do you see a need for patient materials on physical activity/PAP in other languages?

o Yes (1)
o No (2)

## Q28

Have you used the national short version on physical activity in your work?

o Yes (1)
o No, but I know it exists (2)
o No, I didn't know it existed (3)

## Q29

Would agreements between Region JH and activity providers (e.g., gyms, sports clubs) facilitate your ability to prescribe PAP?

o Yes (1)
o To some extent (2)
o Doubtful (3)
o No (4)

## Q30

Most patients who receive PAP do self-directed activities (e.g., home training, walks). Your region is a rural area, which makes collaboration with providers challenging. Would a list of suggested self-activities help you prescribe PAP?

o Yes (1)
o To some extent (2)
o Doubtful (3)
o No (4)

## Q31

Your region has great potential to increase PAP prescriptions. In your view, what are the main barriers in your practice? (Select up to 4)

▢ Lack of knowledge among staff (1)
▢ General time constraints (2)
▢ Unclear purpose of PAP (3)
▢ Patient unwillingness (4)
▢ Lack of leadership support (5)
▢ Lack of information about routines (6)
▢ Technical barriers (7)
▢ Lack of financial incentives (8)
▢ No opinion (9)

## Q32

Please comment on other barriers that make PAP work difficult in your practice:

________________________________________________________________

## Q33

Which of the following measures do you believe would most effectively increase PAP prescriptions in your practice? (Select up to 4)

▢ Train more staff in PAP method (1)
▢ Delegate follow-up to someone else (2)
▢ Increase public awareness (3)
▢ Leadership should prioritize PAP (4)
▢ Improve access to information (5)
▢ Simplify prescription/documentation systems (6)
▢ Improve collaboration (7)
▢ Provide financial incentives (8)
▢ No opinion (9)

## Q34

Please comment on other actions that could support PAP work in your practice:

________________________________________________________________

## Q35

Compared to today, to what extent would you like to work with physical activity counseling in your job?

o Much more (1)
o Somewhat more (2)
o Same amount (3)
o Somewhat less (4)
o Much less or not at all (5)

## Q36

Would you recommend PAP prescription as a method to (new) colleagues?

o Yes (1)
o Unsure (2)
o No / I have no experience with PAP (3)

## Q37

Is there anything else you would like to add?

________________________________________________________________

## Q38

Would you be willing to be interviewed (10–20 min) when the results are compiled? If so, please leave your contact info (mobile or email):

________________________________________________________________
