# Supplementary material for: Barriers and implementation strategies for physical activity on prescription (PAP): healthcare personnel and management perspectives in Sweden—An explanatory sequential study design
Source: BMC Prim Care. 2025 Oct 6;26:302. doi: 10.1186/s12875-025-03038-y (PMC12502212; doi:10.1186/s12875-025-03038-y)
Supplement: Supplementary file 3 — Supplementary Material 3. [file 12875_2025_3038_MOESM3_ESM.docx]

# Questionnaire Survey Managers – English Translation

## Q1

By answering this questionnaire, you confirm that you have read the study information and consent to participate in the study.

## Q2

Gender identity

o Woman (1)
o Man (2)
o Non-binary (3)
o Prefer not to say (4)

## Q3

Which type of practice do you work in?

o Health center (financed by taxes) or a private health center (1)
o Psychiatry, adult or child rehabilitation (2)

## Q4

How do you assess your knowledge of the PAP method for:

Very good (1) / Good (2) / Less good (3) / Not at all good (4)

Promoting health (1)

Preventing certain diseases (3)

## Q5

What is your opinion of the research evidence for PAP as a method to promote health?

o Very strong evidence (1)
o Strong evidence (2)
o Limited evidence (3)
o No evidence (4)
o No opinion (5)

## Q6

What is your opinion of the research evidence for PAP as a treatment or part of treatment for certain diseases?

o Very strong evidence (1)
o Strong evidence (2)
o Limited evidence (3)
o No evidence (4)
o No opinion (5)

## Q7

How do you perceive the prioritization of PAP work for your employees in your practice?

o High priority (1)
o Moderate priority (2)
o Low priority (3)
o No opinion (4)

## Q8

Please motivate your answer:

________________________________________________________________

## Q9

Do you know who is actively working with PAP at your workplace?

o Yes, good knowledge (1)
o Some knowledge (2)
o No knowledge (3)
o No one is actively working with PAP (4)

## Q10

Are you aware of the national guidelines on physical activity?

o Yes (1)
o No (2)

## Q11

Your regin has strong development potential to increase PAP prescriptions. In your opinion, what are the main obstacles in your organization? (Select up to 4)

▢ Lack of knowledge among healthcare staff (1)
▢ General time constraints (e.g., in patient consultations, documentation, learning the method) (2)
▢ Unclear purpose of PAP (3)
▢ Patients unwillingness/are not receptive for PAP (4)
▢ Lack of knowledge/interest from management and leadership to work with PAP (5)
▢ Lack of information about routines related to PAP in the region (6)
▢ Technical barriers (complicated record system, not user-friendly for this purpose) (7)
▢ Lack of financial incentives for number of PAPs prescribed (8)
▢ No opinion (9)

## Q12

Please comment on other barriers in your organization that hinder PAP work:

________________________________________________________________

## Q13

Which of the following actions do you believe would be most effective in increasing PAP prescriptions in your practice? Select the most effective according to your opinion. (Up to 4)

▢ Train more healthcare staff in the PAP method (1)
▢ Delegate the follow-up of PAP to someone other than the prescriber (2)
▢ Increase awareness of physical activity/PAP among the public (3)
▢ Management and leadership need to prioritize PAP (4)
▢ Improve access to PAP information for staff (e.g., on the regional website) (5)
▢ Simplify systems for prescription and documentation (6)
▢ Increase local/regional collaboration on PAP (7)
▢ Financial incentives for number of PAPs (8)
▢ No opinion (9)

## Q14

Please comment on other actions that could support PAP work in your organization:

________________________________________________________________

## Q15

Compared to today, to what extent would you like your staff to work with physical activity counseling in their work?

o Much more (1)
o Somewhat more (2)
o Same extent (3)
o Somewhat less (4)
o Much less or not at all (5)

## Q16

Final question. Please provide examples of what you could contribute to intensify PAP work among your staff:

________________________________________________________________

## Q17

Would you be willing to be interviewed (10–20 min) in March 2024 when results are compiled? If so, please leave your contact details (phone or email):

________________________________________________________________
